# Lnc-AL445665.1–4 may be involved in the development of multiple uterine leiomyoma through interacting with miR-146b-5p

**DOI:** 10.1186/s12885-019-5775-1

**Published:** 2019-07-18

**Authors:** E. Yang, Luqi Xue, Zhengyu Li, Tao Yi

**Affiliations:** 1 Department of Gynecology and Obstetrics, West China Second University Hospital, Sichuan University, Chengdu, 610041 Sichuan People’s Republic of China; 2 Key Laboratory of Obstetrics and Gynecologic and Pediatric Diseases and Birth Defects of Ministry of Education, West China Second Hospital, Sichuan University, Chengdu, 610041 Sichuan People’s Republic of China

**Keywords:** Multiple uterine leiomyoma, Solitary uterine leiomyoma, Long non-coding RNAs, microRNAs

## Abstract

**Background:**

The clinical behaviors and cytogenetics of solitary uterine leiomyomas (SUL) and multiple uterine leiomyomas (MUL) vary, which greatly affects the choice of treatments for reproductive-aged patients with leiomyomas. Our previous study demonstrated that a series of microRNAs, including miR-146b-5p, are dysregulated and play important roles in the development of SUL and MUL. Long non-coding RNAs (lncRNAs) can participate in the pathogenesis of several diseases by regulating the expression of microRNAs; however, their roles in regulating miR-146b-5b and in the pathology of leiomyomas are unclear.

**Methods:**

Pair-matched uterine leiomyoma and adjacent normal myometrium tissue samples were collected from 37 patients with leiomyomas, including 15 with SUL and 22 with MUL. Six paired samples (three SUL and three MUL samples) were used for lncRNAs microarray analysis. Targeted lncRNAs were selected by bioinformatics analysis, and were verified by quantitative reverse transcription-polymerase chain reaction (qRT-PCR) and a dual-luciferase reporter assay. Growth curve analysis and qRT-PCR were used to evaluate the effect of silencing the lncRNA lnc-AL445665.1–4 on cell proliferation and miR-146b-5p expression, respectively.

**Results:**

There were 245 up-regulated and 243 down-regulated lncRNAs in SUL, and 119 up-regulated and 447 down-regulated lncRNAs in MUL. Fifty-five of the selected lncRNAs were predicted to target miR-146b-5p, which is up-regulated in SUL and down-regulated in MUL. Four lncRNAs were selected after Venn diagram analysis showing common dysregulation in the three groups. Lnc-AL445665.1–4 was selected for further exploration. qRT-PCR showed that lnc-AL445665.1–4 expression was significantly up-regulated in MUL compared with SUL in an additional 12 and 19 paired SUL-normal and MUL-normal samples, respectively. The dual-luciferase reporter assay demonstrated the presence of binding sites on lnc-AL445665.1 for miR-146b-5p. Silencing lnc-AL445665.1–4 not only inhibited cell proliferation but also negatively regulated the expression of miR-146b-5p.

**Conclusions:**

Our results suggest that lnc-AL445665.1–4 may be involved in the development of MUL by interacting with miR-146b-5p. Further investigation of the roles of lncRNAs and miRNAs may help to optimize the clinical management of leiomyoma patients. Lnc-AL445665.1–4 could be a novel target for genetic therapy or serve as a biomarker for predicting the recurrence of MUL in patients that have undergone myomectomy.

**Electronic supplementary material:**

The online version of this article (10.1186/s12885-019-5775-1) contains supplementary material, which is available to authorized users.

## Background

Uterine leiomyoma (UL), commonly known as fibroids, originates from the myometrium, and is the most common benign tumor in reproductive-aged females [1]. In the USA, ULs affect at least 70% of women by the age of 50, making it the most common indication of hysterectomy. Therefore, ULs are becoming a public health problem with an increasing financial burden [2, 3]. The majority of patients with leiomyomas are asymptomatic; however, patients with symptomatic leiomyomas experience bleeding, abdominal pain, pressure, pregnancy wastage, or even infertility. Clinically, UL could be manifested as a solitary or multiple form. Solitary uterine leiomyoma (SUL) rarely reoccurs once it is removed, whereas women, especially those of reproductive age, with multiple uterine leiomyoma (MUL) have a higher prevalence of family history or experience menarche at a younger age [4, 5]. These clinical observations suggest that the underlying pathogenesis between MUL and SUL likely differs. Thus, it is necessary to explore these differences in the pathogenesis between SUL and MUL, and identify specific molecules that are differentially expressed in the two conditions so as to further optimize clinical management.

With increasing research efforts focused on the epigenetic causes of human diseases in recent decades, non-coding RNAs have gradually attracted public attention. Long non-coding RNAs (lncRNAs), over 200 nucleotides in length, are not considered to have protein-coding potential. Although the specific functions of the majority of lncRNAs identified to date remain to be uncovered, lncRNAs appear to participate in every biological process. LncRNAs can act as decoys [6], scaffolds [7], or guides [8] to regulate gene expression at epigenetic, transcriptional, and post-transcriptional levels. Accumulating evidence points to a role of lncRNAs in various diseases and in pathogenic processes such as proliferation, apoptosis, and metastases [9–13]. However, there are few studies on the role of lncRNAs in the pathogenesis of UL.

Recently, accumulating research has proven that microRNAs (miRNAs) play important roles in the pathogenesis of UL [14]. In our previous study, we screened out a series of miRNAs, including miR-146b-5p, that were significantly up-regulated in SUL and down-regulated in MUL, demonstrating a role in the development of UL. In addition, several lines of evidence indicate that lncRNAs act as competing endogenous RNAs to silence miRNAs and further promote or inhibit the development of cancers [15–17]. Thus, the aim of the present study was to identify dysregulated lncRNAs targeted by miR-146b-5p in SUL and MUL and explore their roles in the development of ULs.

## Methods

### Patients and tissue samples

Pair-matched UL and adjacent normal myometrium samples were collected from 37 patients that underwent hysterectomy or myomectomy at West China Second University Hospital, Sichuan University (Chengdu, P.R. China). Six pair-matched leiomyoma and adjacent myometrium samples (three with SUL and three with MUL, respectively) were used for lncRNA microarray analysis. An additional 12 pairs of SUL and 19 pairs of MUL samples were used for further quantitative reverse transcription-polymerase chain reaction (qRT-PCR) analysis. All samples were obtained by experienced gynecologists and confirmed by experienced pathologists after myomectomy or hysterectomy. Patients were all at reproductive age. Patients with MUL had five or more leiomyomas. The samples were frozen in liquid nitrogen and stored at − 80 °C until use. The use of patients’ tissue samples was approved by the Institutional Ethics Committee of Sichuan University, and all patients provided written informed consents.

### LncRNA microarray

The microarray work was performed by OE Bio-Tech (Shanghai, China). The Affymetrix Human OElncRNA Array was used in this experiment. Total RNAs were quantified by a NanoDrop ND-2000 spectrophotometer (Thermo Scientific) and the RNA integrity was assessed using Agilent Bioanalyzer 2100 (Agilent Technologies). Genesrping software (version 13.1; Agilent Technologies) was used to complete the basic analysis. Differentially expressed genes were then identified according to the fold change as well as the *P* value calculated with Student t-test. The threshold for up- and down-regulated genes was a fold change ≥2.0 and a P value ≤0.05.

### Target gene prediction

Target gene prediction was performed with the online software Miranda (http://www.microrna.org/microrna/home.do, v3.3a, microRNA Target Scanning Algorithm, a software written by Anton Enright). The detailed information is provided in Tables 1 and 2.

**Table 1:** Basic information of selected lncRNAs

LncRNA ID	FC (abs)	Regulation	Chromosome	Strand	Start	End	Database
lnc-AL445665.1–4	3.3	down	chr9	positive	65,218,522	65,219,575	NONCODE
lnc-GUCY1A3–1	3.01	down	chr4	positive	155,734,447	155,737,062	NONCODE
lnc-LHFPL3–5	2.04	down	chr1	negative	210,231,456	210,234,121	Ensembl
lnc-RP4-725G10.1.1–8	3.65	down	chr8	negative	17,643,794	17,800,917	Ensembl

Note: FC (abs): absolute value of fold change

**Table 2:** The information of lncRNAs targeting by miR-146b-5p

LncRNAs	Targeting sites
lnc-GUCY1A3–1	Query: 3′ ucGGAUACCUUAAGUCAAGAGu 5’
	::|:| ||:||:||||:||
	Ref: 5′ tcTTTGTTGAGTTTAGTTTTCt 3’
lnc-AL445665.1–4	Query: 3′ ucgGAUACCUU--AAGUCAAGAGu 5’
	|||| || |||||||||:
	Ref: 5′ agaCTATTAAATGTTCAGTTCTTc 3’
lnc-LHFPL3–5	Query: 3′ ucggaUACCU-UAAGUCAAGAgu 5’
	|| || | ||||||||
	Ref: 5′ gagtaATAGACAGTCAGTTCTga 3’
lnc-RP4-725G10.1.1–8	Query: 3′ ucggaUACCU--UAAGUCAAGAGu 5’
	||| | || |||||||:
	Ref: 5′ cgaaaATGCAAGATGCAGTTCTTt 3’

### RNA isolation and qRT-PCR

Total RNA extraction either from tissues or cells was performed using Trizol reagent (Invitrogen). The yield of RNA was determined using a NanoDrop 2000 spectrophotometer (ThermoScientific, USA), and the integrity was evaluated using agarose gel electrophoresis stained with ethidium bromide. Each RT reaction was performed in a GeneAmp PCR System 9700 (Applied Biosystems, USA). qPCR was performed using a LightCycler 480 II Real-time PCR instrument (Roche, Swiss). The primer sequences are summarized in Table 3. The relative expression levels of lncRNAs and miR-146b-5p were calculated by the 2^−△CT^ method [18]. LncRNA expression levels were normalized to the level of *ACTB*, while miR-146b-5p expression levels were normalized to that of *U6*. Each experiment was performed in triplicate and each measure was also determined in triplicate.

**Table 3:** Primer sequences for quantitative reverse transcription polymerase chain reaction

Gene	Forward primer	Reverse primer
lnc-LHFPL3–5	5′ ACTTCTACTATTGATGCAGTCC 3’	5′ AAGTTCAGAACTGACTGTCTAT 3’
lnc-GUCY1A3–1	5′ GCATCCAGACACTGCTTAATC 3’	5′ GCAAGGAGGTGAAGGAATAG 3’
lnc-AL445665.1–4	5′ ATTCTGATTCCCTGTGTATCCA 3’	5′ CTGTGACAGAAATAGGAATGCC 3’
lnc-RP4-725G10.1.1–8	5′ GCTCAGGACTTTCACTGATT 3’	5′ GTTGTTTCAACTTCTTTCCCAC 3’
ACTB	5′ CCATCATGAAGTGTGACG 3’	5′ GCCGATCCACACGGAGTA 3’
miR-146b-5p	5′ GCGCAGTGAGAACTGAATTCCA 3’	5′ AGTGCGTGTCGTGGAGTCG3’
U6	5′ CGATACAGAGAAGATTAGCATGGC 3’	5′ AACGCTTCACGAATTTGCGT 3’

### Dual-luciferase reporter assay

A firefly luciferase reporter plasmid (psiCHECK-lncAL445665.1–4, psiCHECK-lncAL445665.1–4-mut,psiCHECK-lnc-LHFPL3–5,psiCHECK-lnc-LHFPL3–5-mut) and a luciferase vector (PsiCHECK-2Vector, Promega) plus small RNAs (miR-146b-5p and its negative control) were co-transfected into HEK293T cells with Lipofectamine 2000 (Invitrogen). Firefly luciferase activity was normalized to *Renilla* activity, and the result was expressed as a relative value. All the procedures were followed the manufacturer’s instructions.

### Cell culture and transfection

We used the endometrial cell line Ishikawa instead of primary UL cells because the latter were difficult grow. Moreover, a previous study [19] showed that miR-146b-5p is involved in the development of Ishikawa cells. Ishikawa cells were cultured in Dulbecco’s modified Eagle’s medium (Gibco, Carlsbad, CA, USA) mixed with 10% fetal bovine serum (Gibco) and 1% penicillin and streptomycin (Gibco). All cells were cultured in a 37 °C incubator containing 5% CO_2_. The si-lncAL445665.1–4 and its negative control (si-NC), and miR-146b-5p mimics and corresponding negative controls were all purchased from RiboBio (Guangzhou, China). Transfection was carried out using Lipofectamine 3000 (Invitrogen) according to the manufacturer’s instructions. The transfection effects of si-lncAL445665.1–4 (100 nM) and miR-146b-5p mimics (200 nM) were examined by qRT-PCR using RNA extracted 48 h after transfection. Cell viability was determined at 24, 36, 48, and 72 h after transfection using si-lncAL445665.1–4, si-NC, miR-146b-5p-mimic, and miR-146b-5p-mimicNC-transfected Ishikawa cells.

### MTT assay

Cellular proliferation assay was performed by MTT assay. All procedures were followed the manufacturer’s instructions and previous studies. The optical density value was measured by a Varioskan Flash (ThermoScientific) at a wavelength of 490 nm.

### Statistical analysis

Results are expressed as mean ± SEM. GraphPad Prism (GraphPad Software Inc.) was applied for data analysis with all data assessed for a normal distribution and equal variance. Statistical comparisons among different groups were performed by one-way analysis of variance. Differences between two groups were evaluated by Student’s *t-*test. *P* < 0.05 was considered statistically significant.

## Results

### Four lncRNAs were selected from lncRNAs microarray analysis

**Fig. 1:**
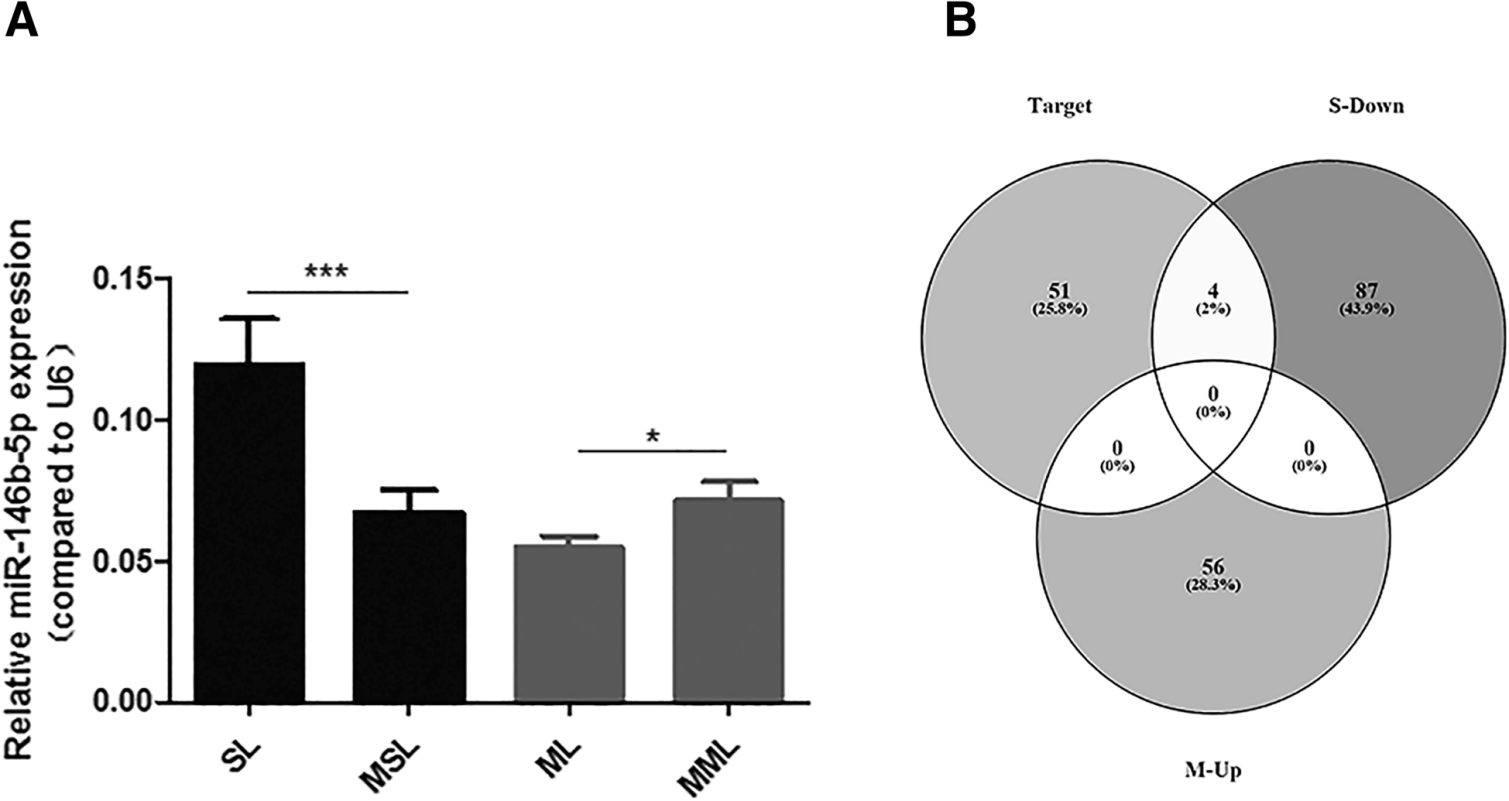
**a** The relative expression of miR-146b-5p in our previous findings, miR-146b-5p was extremely significantly up-regulated in SUL and significantly down-regulated in MUL compared with adjacent myometrium. **b** A Venn analysis of three groups: lncRNAs targeted with miR-146b-5p, lncRNAs down-regulated in SUL and lncRNAs up-regulated in MUL. Four lncRNAs were selected after Venn analysis. SL: solitary uterine leiomyoma, MSL: myometrium of solitary uterine leiomyoma, ML: multiple uterine leiomyoma, MML: myometrium of multiple uterine leiomyoma, S-down: lncRNAs down-regulated in SUL, M-up: lncRNAs up-regulated in MUL. Target: lncRNAs targeting with miR-146b-5p predicted by Miranda

**Fig. 2:**
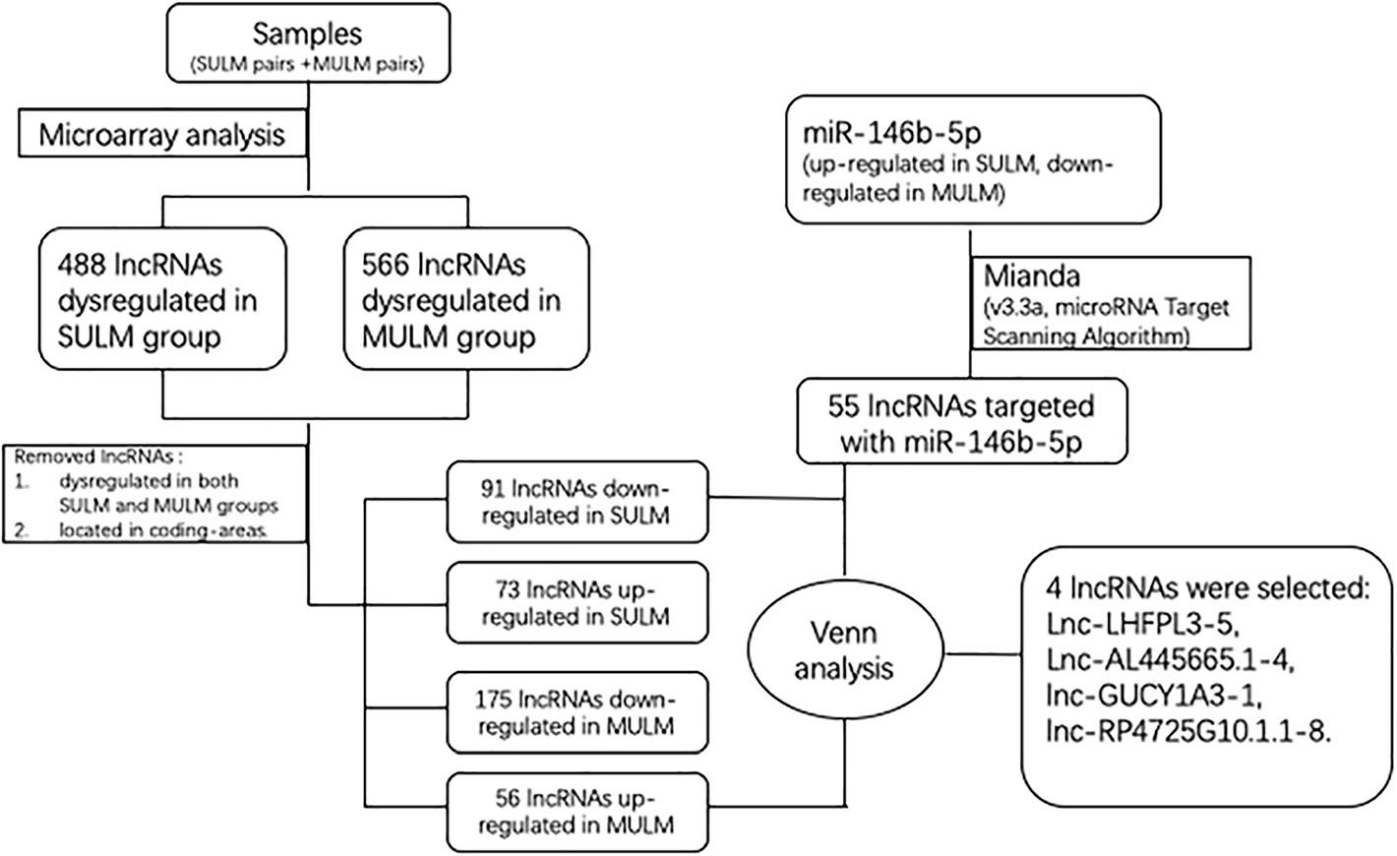
The flow chart of selecting lncRNAs of interest

LncRNAs in paired UL and normal samples were screened by lncRNA microarray analysis. Thousands of dysregulated lncRNAs in our six pairs of leiomyoma tissue samples were obtained, which are presented in Additional file 1: Figure S1A. To narrow the screening region, we removed repetitive lncRNAs and selected lncRNAs dysregulated in the SUL and MUL groups compared to their adjacent normal myometrium samples (paired t-test; threshold for selected genes, *p* < 0.05, fold change ≥2.0. After the preliminary screening, a total of 488 and 566 lncRNAs were found to be dysregulated in the SUL and MUL group compared with the adjacent myometrial groups, respectively (Additional file 1: Figure S1B, C). After removing 43 lncRNAs dysregulated in both the SUL and MUL groups and genes in coding regions, 73 up-regulated and 91 down-regulated lncRNAs specifically expressed in SUL and 56 up-regulated and 175 down-regulated lncRNAs specifically expressed in MUL were selected out (Additional file 2: Figure S2A, 2B). Next, we used Miranda (v3.3a, microRNA Target Scanning Algorithm) to predict the lncRNAs targeted by miR-146b-5p, which predicted 55 targeted lncRNAs. Focusing on the expression of miR-146b-5p, which is up-regulated in SUL and down-regulated in MUL (Fig. 1a), the lncRNAs of interest needed to meet two requirements: targeted by miR-146b-5p, and up-regulated in MUL **or** down-regulated in SUL. As a result, four lncRNAs, lnc-AL445665.1–4, lnc-LHFPL3–5, lnc-GUCY1A3–1, and lnc-RP4725G10.1.1–8, were selected out after Venn analysis in the three groups: 55 lncRNAs were predicted to target miR-146b-5p, and were down-regulated in SUL and up-regulated in MUL (Fig. 1b). The detailed information of these lncRNAs is shown in Tables 1 and 2. All steps of selecting lncRNAs of interest are presented in a flow chart in Fig. 2.

### Lnc-AL445665.1–4 was significantly up-regulated in MUL samples

**Fig. 3:**
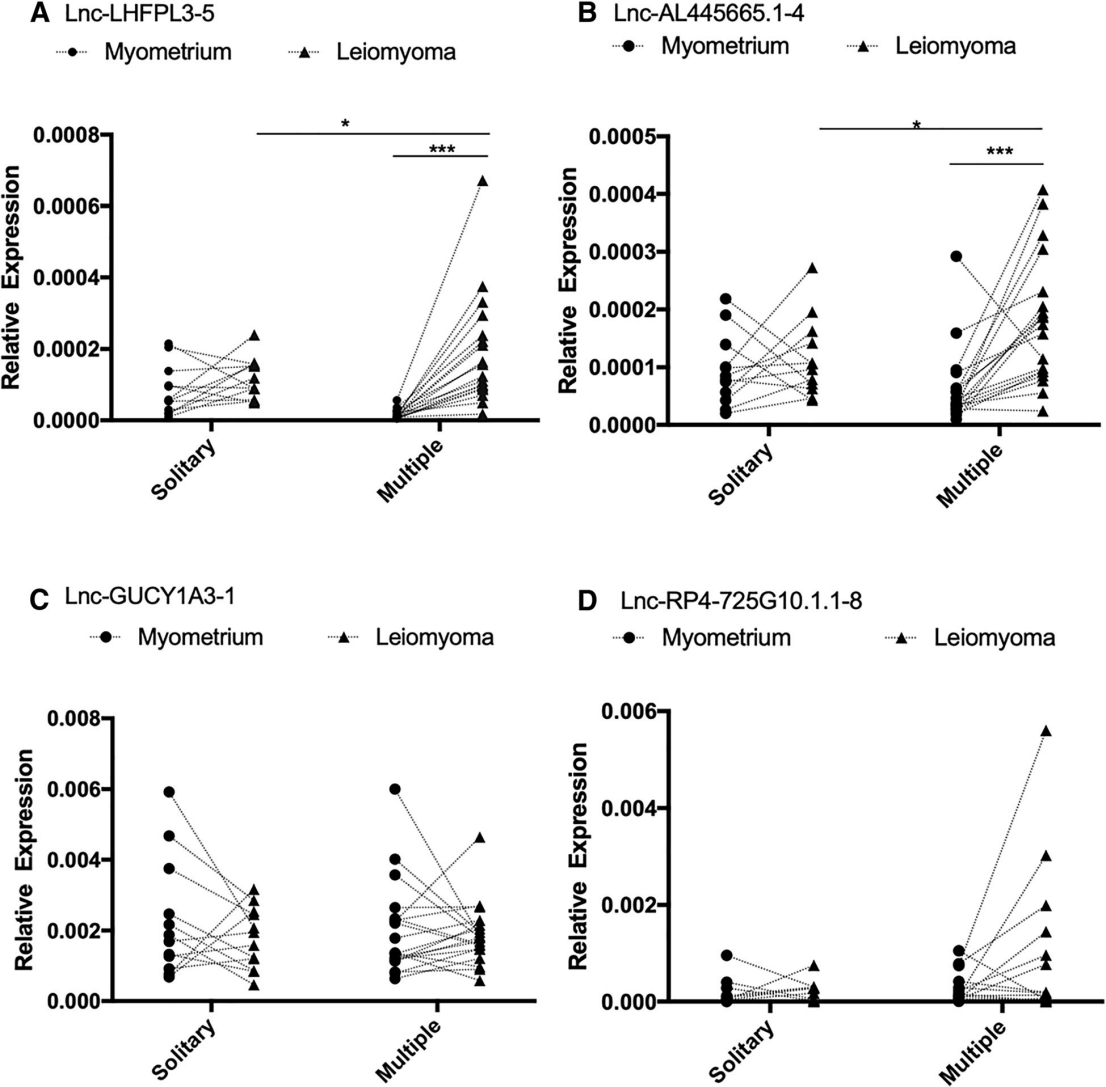
The qRT-PCR results of these four selected lncRNAs. Additional 12 pairs of SUL specimens and 19 pairs MUL specimens were used in qRT-PCR analysis to verify the expression of four lncRNAs. lnc-AL445665.1–4 and lnc-LHFPL3–5 were both significantly up-regulated in MUL. Here, the value of relative expression was 2-ΔCt, ΔCt = Ct (lncRNA)-Ct (ACTB). *p<0.05, ***p<0.001

Next, qRT-PCR was used to verify the expression of these selected lncRNAs, using a separate set of 12 pairs of SUL and 19 pairs of MUL samples. As shown in Fig. 3, the expression of lnc-AL445665.1–4 and lnc-LHFPL3–5 were highly significantly up-regulated in MUL compared to those in the adjacent myometrium (*p* < 0.001), whereas there was no significant difference in the expression of these lncRNAs between SUL and adjacent myometrium samples. Furthermore, these two lncRNAs were significantly up-regulated in the MUL group compared to the SUL group (*p* < 0.05). With respect to the remaining two lncRNAs, there were no significant differences in their relative expression levels in both the SUL and MUL groups in comparison with those of the corresponding control groups (Fig. 3). These results implied that lnc-AL445665.1–4 and lnc-LHFPL3–5 are significantly up-regulated specifically in MUL.

### Lnc-AL445665.1–4 directly targeted miR-146b-5p

**Fig. 4:**
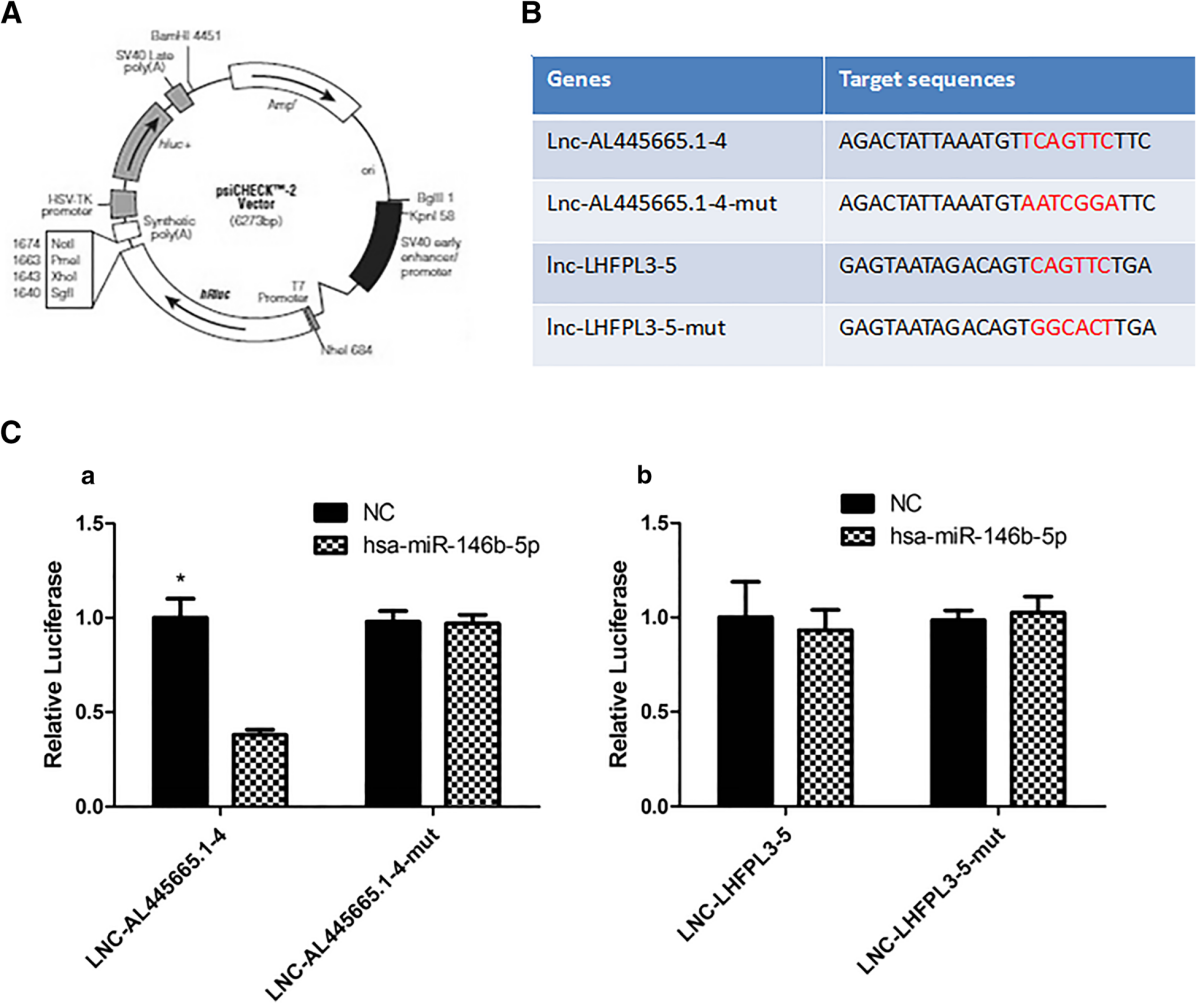
Dual-luciferase reporter assay between lnc-AL445665.1–4, lnc-LHFPL3–5 and miR-146b-5p. **a**: The structure of our luciferase vector: PsiCHECK-2Vector. **b**:The information of two selected lncRNAs and their mutations partial sequences for dual-luciferase reporter assay. **c**(a): the fluorescent expression of psiCHECK-LNC-AL445665.1–4 could be significantly suppressed by has-miR-146b-5p (*p*<0.05); the fluorescent expression of psiCHECK-LNC-AL445665.1–4-mut could not be suppressed by has-miR-146b-5p (*p*>0.05). C(b): has-miR-146b-5p could not suppress neither the fluorescent expression of psiCHECK-LNC-LHFPL3–5 nor that of psiCHECK-LNC-LHFPL3–5-mut (*p*>0.05)

To determine the existence of binding sites between lnc-AL445665.1–4, lnc-LHFPL3–5, and miR-146b-5p, a dual-luciferase reporter assay was performed. The results showed that the luciferase activity of psiCHECK-LNC-AL445665.1–4 was significantly suppressed by miR-146b-5p (*p* < 0.05), while no apparent alteration of the luciferase activity of psiCHECK-LNC-AL445665.1–4-mut, psiCHECK-LNC-LHFPL3–5, and psiCHECK-LNC-LHFPL3–5-mut was observed, indicating that only lnc-AL445665.1–4 directly targets miR-146b-5p (Fig. 4).

### Silencing lnc-AL445665.1–4 influenced the proliferation of Ishikawa cells and increased the expression level of miR-146b-5p

**Fig. 5:**
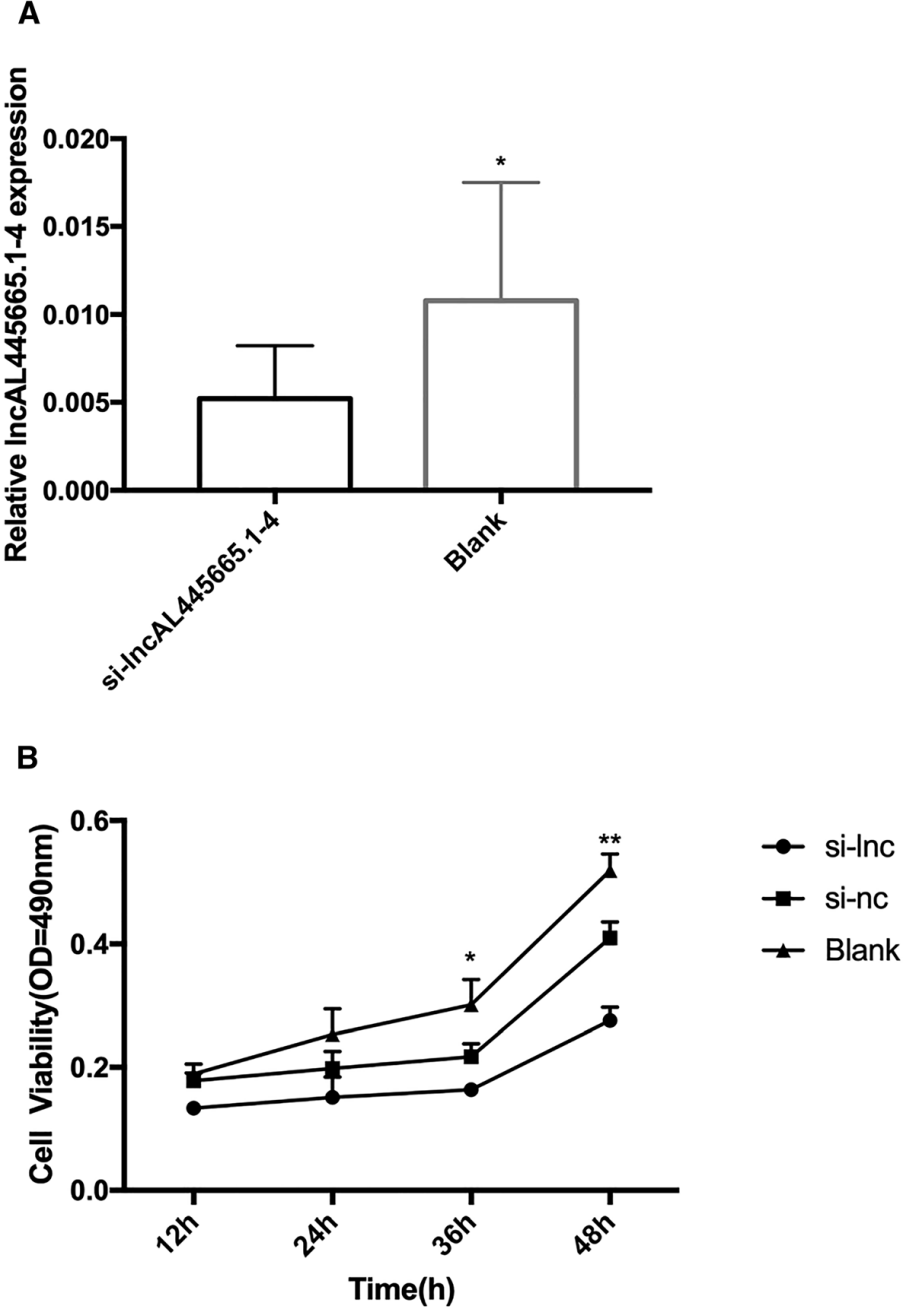
Silencing lnc-AL445665.1–4 influenced the proliferation of Ishikawa cells. **a** siRNA was used to silence the expression of lnc-AL445665.1–4 in Ishikawa cells. **b** 12,24, 36 and 48 h after transfection, the viability of Ishikawa cells was analyzed. Comparing with si-NC and blank groups, the proliferation of Ishikawa cells became significantly slower in si-lnc-AL445665.1–4 group

**Fig. 6:**
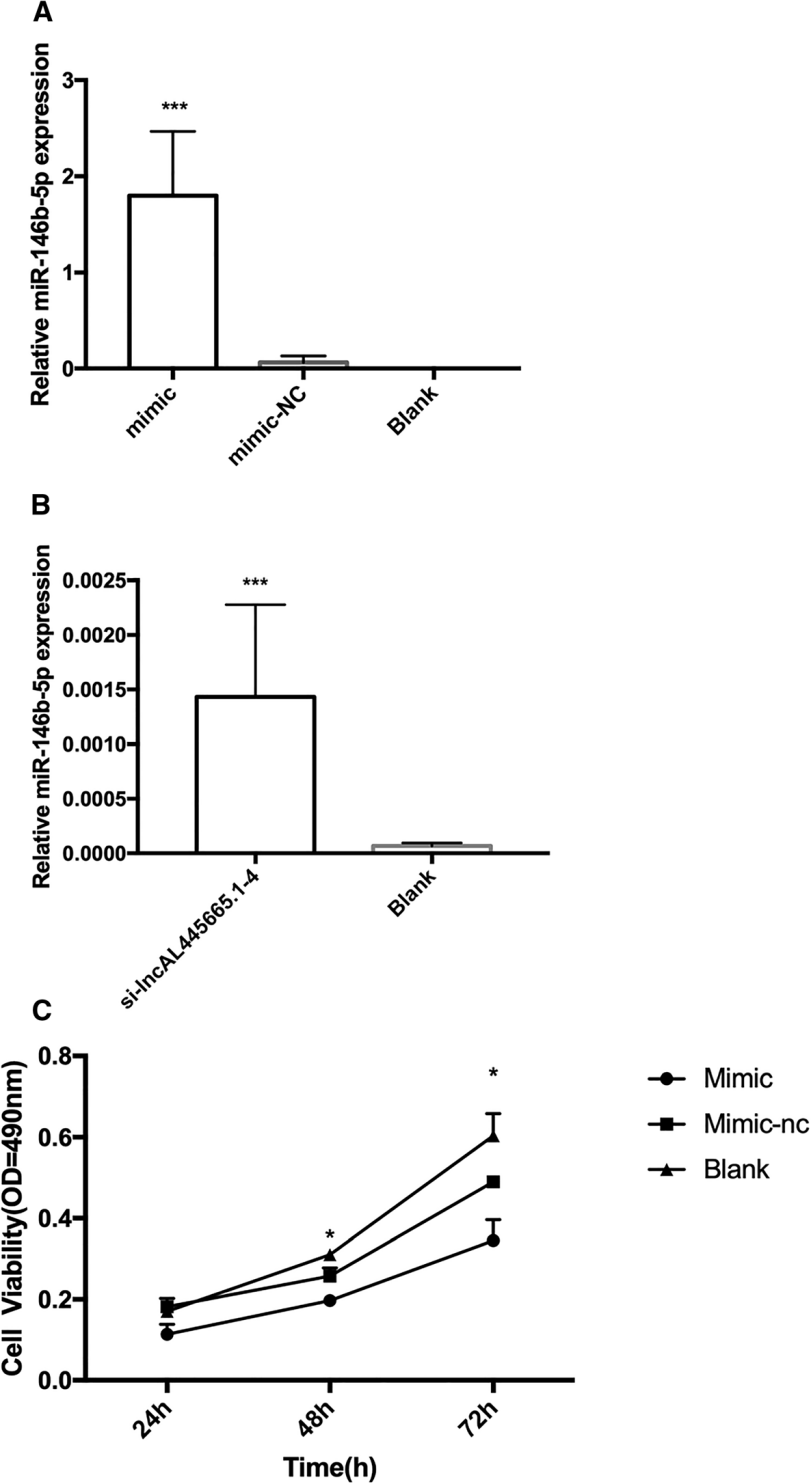
Enforced expression of miR-146b-5p negatively influenced the proliferation of Ishikawa cells. **a** Forty-eight hours after transfection, the result of qRT-PCR showed that miR-146b-5p significantly over expressed. **b** Silencing lnc-AL445665.1–4 increased the expression of miR-146b-5p. Forty-eight hours after infection, the expressions of lnc-AL445665.1–4 and miR-146b-5p were analyzed. **c** Cell viability tested by MTT showed that the proliferation of Ishikawa cells was significantly slower after 24, 48 and 72 h transfection

Finally, we explored the function of lnc-AL445665.1–4 in vitro using Ishikawa cells. The proliferation rate of Ishikawa cells was reduced and miR-146b-5p expression was significantly up-regulated in Ishikawa cells after silencing lnc-AL445665.1–4. Moreover, overexpression of miR-146b-5p also reduced the proliferation rate of Ishikawa cells (Figs. 5 and 6). These data implied that lnc-AL4455665.1–4 may be involved in the development of MUL through interaction with miR-146b-5p.

## Discussion

UL is the most common form of benign genital tumor in reproductive-aged females. The pathogenesis of UL is currently considered to result from several genetic and epigenetic mechanisms, along with regulation by sex steroids, cytokines, chemokines, and extracellular matrix mediators [20–22]. However, the exact processes of these mechanisms remain unclear. Clinically, the different behaviors between SUL and MUL causes confusion, suggesting that the pathogenesis between these two kinds of ULs is different. In our previous study, we identified some miRNAs that are differentially expressed between MUL and SUL, including miR-146b-5p, which was significantly down-regulated in MUL and up-regulated in SUL. In the present study, we found that the lncRNA lnc-AL445665.1–4 was significantly up-regulated in MUL and targeted miR-146b-5p. Moreover, silencing lnc-AL445665.1–4 in vitro could negatively influence the growth of Ishikawa cells and promote the expression of miR-146b-5p. In addition, overexpression of miR-146b-5p also inhibited the growth of Ishikawa cells. Taken together, our results suggest that lnc-AL445665.1–4 may promote the development of MUL by negatively regulating miR-146b-5p.

Our study is not the first to explore the differences between SUL and MUL. In the early 2010s, a study demonstrated that Caucasian females with MUL had a higher prevalence of a family history, earlier age of menarche, lower parity, higher percentage of smoking, lower prevalence of the *CYP17A1* AA genotype, and lower *CYP17A1* A allele frequency than healthy females; however, these factors were not significant among patients with SUL [5]. Mutation of *MED12* and rearrangement of *HMGA2* are the most common genetic subtypes in UL according to a recent study [23]. Moreover, the mutation of *MED12* was shown to be more common in MUL patients, while the mutation of *HMGA2* was largely observed in SUL cases, and no single patient exhibited both mutations simultaneously [24, 25]. As UL is a sex hormone-induced tumor, the difference between SUL and MUL also involves the expression of estrogen receptor (ER), with lower expression of ERα and higher expression of ERβ in MUL than in SUL [26].

These previous findings combined with our present results highlight different pathogenic mechanisms between MUL and SUL. We have identified some novel specific lncRNAs and miRNAs that are differentially expressed in SUL and MUL. If more specific genes or molecules are identified in future similar investigations, they could be used as markers to guide the clinical treatment of leiomyomas. For patients with MUL, the option of myomectomy can preserve the uterus, but the risk of recurrence is higher than for patients with SUL that undergo myomectomy. Alternatively, the option of radical hysterectomy avoids the risk of recurrence but this comes at a cost of the chance to become pregnant for reproductive-aged females. Thus, identification of the molecules that are specifically expressed and involved in the pathogenesis of MUL can offer new approaches for the prevention and treatment of MUL. In addition, these molecules could be regarded as risk factors for building a model to predict the recurrence of MUL and further serve as a guide to optimize clinical decisions for patients with leiomyoma.

Several studies have suggested that miR-146b-5p plays an important role in the development of certain diseases, including cancers. Overexpression of miR-146b-5p could inhibit the growth of glomerular mesangial cells and reduce the levels of IL6 and IL8 in lupus nephritis [27]; suppress cell proliferation, migration, and invasion in non-small cell lung cancer [28]; promote cell growth, invasion, and glycolysis in colorectal cancer [29]; and slow down the growth of Ishikawa cells in the present study.

Numerous studies have proven that lncRNAs and the interaction between lncRNAs and miRNAs play important roles in both physiological and pathological mechanisms. Lnc-DC could regulate human conventional dendritic cells [30], linc-MD1 was shown to be involved in governing the timing of muscle differentiation by sponging miRNAs in human myoblasts [15], CHRF regulated cardiac hypertrophy by suppressing miR-489 and releasing Myd88 [17], and lncRNA-ATB promoted the metastasis and invasion of hepatocellular carcinoma cells by sponging miR-200 family and then induced epithelial-mesenchymal transition [10]. Moreover, HOXA11-AS could promote renal cancer cells growth and invasion by modulating the miR-146b-5p–MMP16 axis [31]. Our result showed that silencing lnc-AL445665.1–4 could increase the expression of miR-146b-5p. Therefore, we speculate that there may exist some mechanism by which lnc-AL445665.1–4 controls the expression of miR-146b-5p and then further regulates the development of MUL. However, further detailed investigations are needed to uncover the exact mechanism underlying the interaction of lnc-AL445665.1–4 and miR-146b-5p in the pathogenesis of MUL.

There are some limitations of our current study. First, the number of tissue samples for qRT-PCR analysis was limited. Second, we used Ishikawa cells instead of primary leiomyoma cells to explore the function of lnc-AL445665.1–4 because of the challenge in growing the primary cells.

## Conclusions

In summary, our present study provides the first evidence that the expression level of lnc-AL445665.1–4 is increased in MUL compared to that in SUL. Functional analyses further showed that lnc-AL445665.1–4 may promote the development of MUL by inhibiting the expression of miR-146b-5p. The exact mechanism of this interaction between lnc-AL445665.1–4 and miR-146b-5p will be investigated in our further research. Overall, our result expands current understanding of the pathogenesis of leiomyoma at the lncRNAs-miRNA level.

## Additional files

Additional file 1: **Figure S1** Preliminary screening results of dysregulated genes in SUL and MUL. A. The result of dysregulated lncRNAs in six pairs of leiomyoma specimens compared with adjacent myometrium in lncRNAs microarray analysis. B and C: The volcano plots of dysregulated lncRNAs in SUL and MUL respectively, according to the result showed in graph A, we selected the lncRNAs which dysregulated in all three pairs of SUL and MUL groups respectively, statistical method was paired t test (SUL 1,2,3 VS MSUL1,2,3; MUL 1,2,3 VS MMUL 1,2,3, the threshold for selected genes were p<0.05 and fold change value>2.0). As a result, there were 488 and 566 dysregulated lncRNAs in SUL and MUL group respectively. S: solitary uterine leiomyomas vs myometrium of solitary uterine leiomyomas; M: multiple uterine leiomyomas vs myometrium of multiple uterine leiomyomas. (TIF 2721 kb)

Additional file 2: **Figure S2** The clustering maps of dysregulated lncRNAs in SUL (A) and MUL (B). (TIF 1889 kb)

## Data Availability

The datasets used and/or analysed during the current study are available from the corresponding author on reasonable request.

## Abbreviations

LncRNAs: Long non-coding RNAs
microRNAs: miRNAs
MUL: Multiple uterine leiomyoma
qRT-PCR: Quantitative reverse transcription PCR
RT: Reverse transcription
SUL: Solitary uterine leiomyoma
UL: Uterine leiomyoma
