# Supplementary figures and images for: Lnc-AL445665.1–4 may be involved in the development of multiple uterine leiomyoma through interacting with miR-146b-5p
Source: BMC Cancer. 2019 Jul 18;19:709. doi: 10.1186/s12885-019-5775-1 (PMC6639973; doi:10.1186/s12885-019-5775-1)

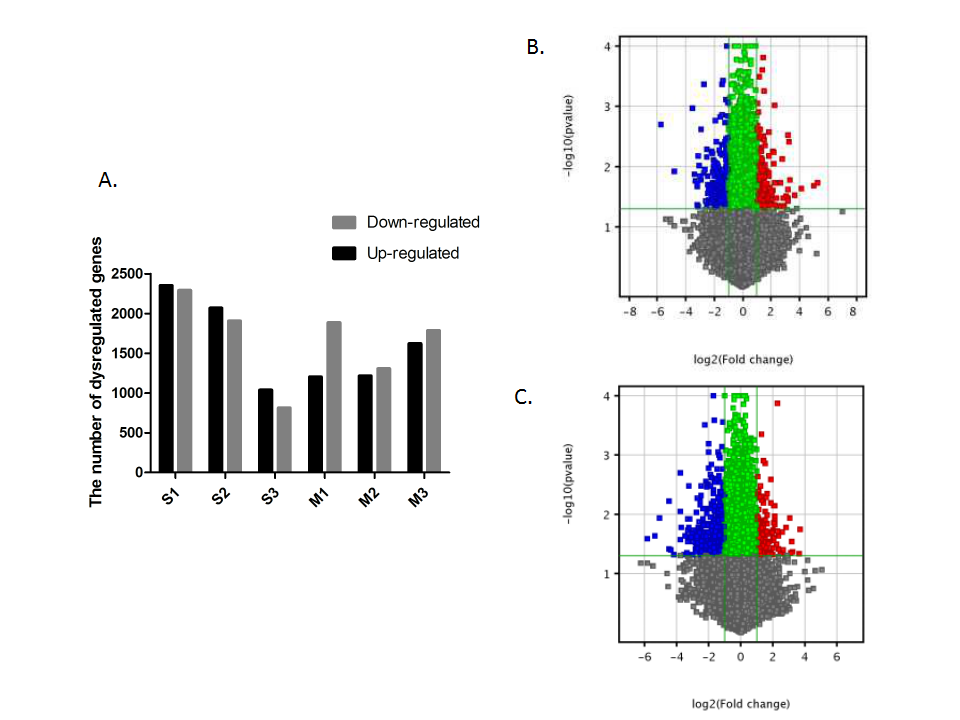

Supplement: Supplementary file 1 — Figure S1 Preliminary screening results of dysregulated genes in SUL and MUL. A. The result of dysregulated lncRNAs in six pairs of leiomyoma specimens compared with adjacent myometrium in lncRNAs microarray analysis. B and C: The volcano plots of dysregulated lncRNAs in SUL and MUL respectively, according to the result showed in graph A, we selected the lncRNAs which dysregulated in all three pairs of SUL and MUL groups respectively, statistical method was paired t test (SUL 1,2,3 VS MSUL1,2,3; MUL 1,2,3 VS MMUL 1,2,3, the threshold for selected genes were p<0.05 and fold change value>2.0). As a result, there were 488 and 566 dysregulated lncRNAs in SUL and MUL group respectively. S: solitary uterine leiomyomas vs myometrium of solitary uterine leiomyomas; M: multiple uterine leiomyomas vs myometrium of multiple uterine leiomyomas. (TIF 2721 kb) [file 12885_2019_5775_MOESM1_ESM.tif]

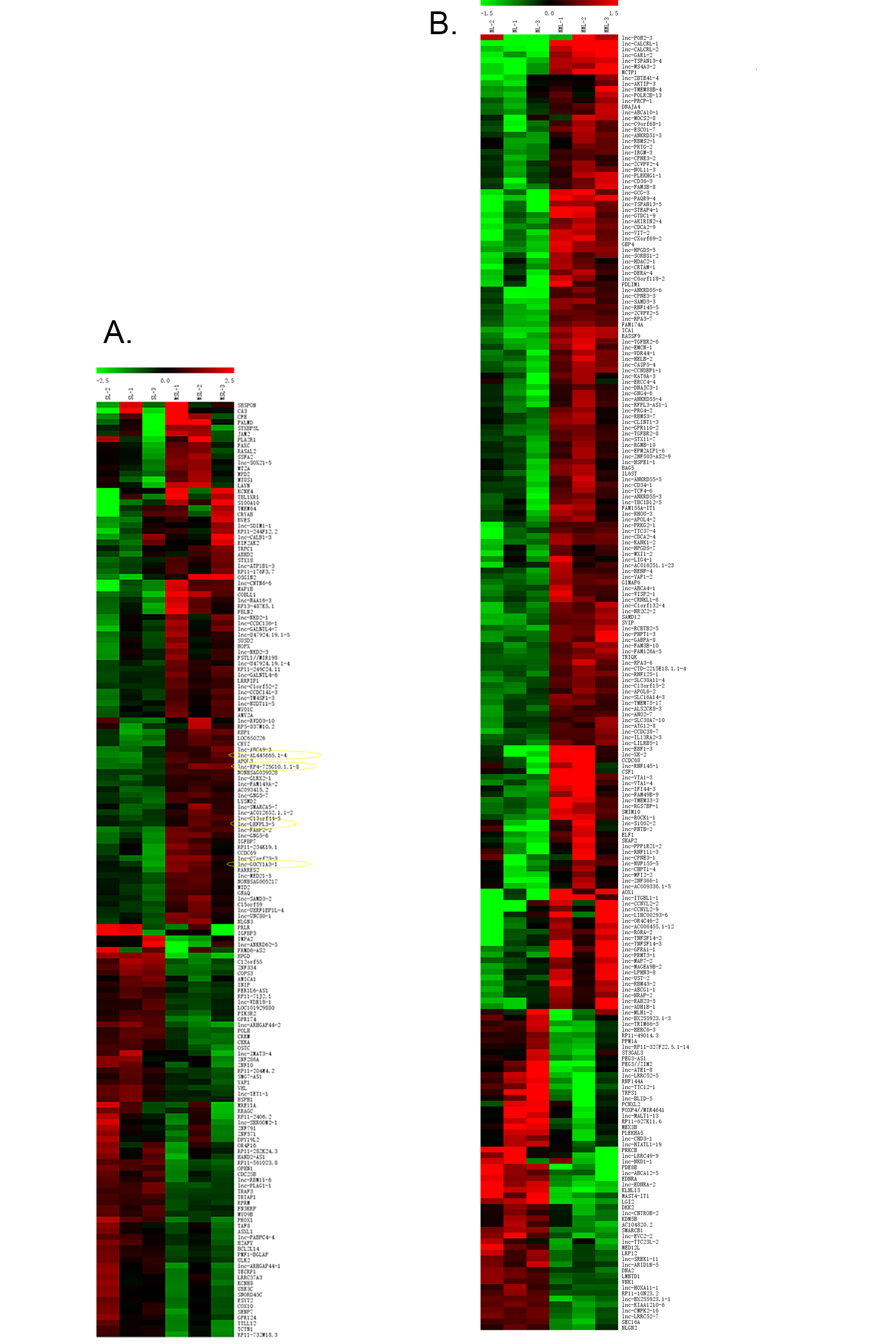

Supplement: Supplementary file 2 — Figure S2 The clustering maps of dysregulated lncRNAs in SUL (A) and MUL (B). (TIF 1889 kb) [file 12885_2019_5775_MOESM2_ESM.tif]
